# Complementation of the Yeast Model System Reveals that *Caenorhabditis elegans* OCT-1 Is a Functional Transporter of Anthracyclines

**DOI:** 10.1371/journal.pone.0133182

**Published:** 2015-07-15

**Authors:** Nicolas Brosseau, Emil Andreev, Dindial Ramotar

**Affiliations:** Maisonneuve-Rosemont Hospital, Research Center, Université de Montréal, department of Medicine, 5415 Boul. de l’ Assomption, Montréal, Québec, Canada; Université de Sherbrooke, Medicine, CANADA

## Abstract

The yeast plasma membrane protein Agp2 belongs to the family of amino acid transporters. It acts as a regulator that controls the expression of several uptake transporter genes such as *DUR3* and *SAM3* encoding two high-affinity polyamine permeases. *agp2Δ* mutants display extreme resistance to several cationic compounds including polyamines, the anticancer agent bleomycin, and cationic antifungal peptides. We propose that Agp2 might be involved in regulating the uptake of other cationic anticancer drugs. To date, an uptake transporter has not been reported for anthracyclines, a family of chemotherapeutic agents that are used for treating adult patients with acute myeloid leukemia. Herein, we develop assay conditions to monitor the uptake of the anthracycline doxorubicin into yeast cells and demonstrate for the first time that Agp2 is required for the drug uptake. Deletion of both the *DUR3* and *SAM3* genes reduced doxorubicin uptake, but not the deletion of either gene alone, while the *agp2Δ* mutant was severely compromised, suggesting that Agp2 controls the drug uptake *via* Dur3 and Sam3 and at least one additional transporter. Overexpression of DUR3 or SAM3 from the endogenous promoter rescued doxorubicin uptake into the *sam3Δdur3Δ* double mutant, consistent with a role for these transporters in the uptake of anthracyclines. We further show by cross-species complementation analysis that expression of the *Caenorhabditis elegans oct-1* gene encoding an organic cation transporter restored full doxorubicin uptake in the *agp2Δ* mutant. Four separate variants of CeOCT-1 derived by substituting the amino acid residues Gln15, Cys31, Gln109 and Lys300 with alanine were stably expressed, but did not mediate doxorubicin uptake into the *agp2Δ* mutant. Moreover, we show that overexpression of CeOCT-1 sensitized parent yeast cells to doxorubicin, suggesting that CeOCT-1 related members might be key transporters to facilitate entry of anthracyclines into human cells.

## Introduction

The yeast *Saccharomyces cerevisiae* plasma membrane protein, Agp2, was initially identified as a transporter for L-carnitine, which serves as a carrier for acetyl-CoA from the peroxisomes to the mitochondria [1]. We subsequently re-isolated the *AGP2* gene by screening the collection of yeast haploid mutants, each deleted for a single gene, and demonstrated that *agp2Δ* mutant is extremely resistant to the anticancer drug bleomycin that acts by damaging the cellular DNA [2]. *agp2Δ* mutants are completely defective in the uptake of a fluorescently labeled form of bleomycin and displayed extremely low levels of bleomycin-induced damage to the DNA [2]. In contrast, overexpression of Agp2 stimulated bleomycin uptake and caused severe damage to the genomic DNA, suggesting that Agp2 indeed functions to allow uptake of bleomycin into the cells [2]. The bleomycin used in these studies contains a polyamine moiety, which raised the possibility that bleomycin entry into the cells might occur because of the polyamine group and that Agp2 would be involved in polyamine uptake. Indeed, we have shown that *agp2Δ* mutants are strikingly resistant to polyamines and completely blocked for the uptake of very low concentrations of spermine and spermidine [3]. While these earlier studies strongly suggest that Agp2 might function as a high affinity transporter for L-carnitine, bleomycin and polyamines, subsequent findings dismissed this notion. These include (i) L-carnitine, even at high concentrations, did not block the uptake of labeled spermidine into the cells or protected parent cells from the cytotoxic effects of polyamines, and (ii) a study by Uemura *et al* documented the existence of two high affinity polyamine transporters, Dur3 and Sam3, which exist on the plasma membrane of yeast cells, although the link to Agp2 was not investigated [4, 5]. More recently, we showed that Agp2 acts as a regulator that prominently controls the expression of the SR kinase gene *SKY1*, as well as several transporter genes including *DUR3*, *SAM3* and *HNM1* that encodes a L-carnitine transporter [5]. Deletion of either the *DUR3* or *SAM3* gene resulted in mutants that exhibit parental level of resistance to polyamines [5]. However, deletion of both genes resulted in double mutants that were resistant to polyamines [5]. The exact nature by which Agp2 regulates these transporter genes remains unclear, although we believe that it acts as a sensor of various cationic compounds in the media and transmits a signal to maintain the expression of many genes including the transporter genes [5]. Consistent with this notion, an independent study revealed that Agp2 is also involved in the uptake of the antifungal drug NaD1, but it is not known which of the transporters regulated by Agp2 is involved in NaD1 uptake [6].

To date, the mode of entry of a number of highly active hydrophilic anticancer drugs into cells is not known [7, 8] One family of these drugs is the anthracyclines that are cationic in nature and include doxorubicin and daunorubicin (DNR), which must be transported into the cells where they act by intercalating with the DNA and block, e.g., the function of DNA topoisomerase leading to cell death [9]. Anthracyclines are used for treating adult patients with acute myeloid leukemia (AML)[10]. This disease is characterized by the rapid expansion of immature blood cells and is the major cause of mortality from hematological malignancies in adults [10]. Importantly, a significant fraction (> 50%) of older AML patients (> 60 yrs) are resistant to chemotherapy with anthracyclines [11–13]. A characteristic mechanism associated with drug resistance is the elevated levels of plasma membrane ABC transporters [14–16]. These include the multidrug resistant efflux pump, MDR1/ABCB1, and the multidrug resistant-associated protein, MRP1, which are known to increase efflux of chemotherapeutic agents thereby allowing tumor (and normal) cells to evade drug-induced cytotoxicity [14, 15]. In fact, drug efflux transporters are known to be upregulated in some AML patients and there is evidence suggesting that one of these efflux pumps ABCB1 can expel anthracyclines from the cells [17]. However, inhibition of ABCB1 with valspodar was rigorously tested and found to provide no improvement for the drug-resistant AML patients [17]. Thus, there must be other mechanisms that when defective would cause resistance to anthracyclines. One such mechanism might involve defects in the entry of DNR into leukemia cells via uptake transporter(s). So far, an uptake transporter system for anthracyclines has not been previously described although in human cells one study claimed that it is the organic cation transporter hCT2, but this transporter has not been further characterized [18].

Since Agp2 regulates the uptake of several positively charged molecules, we tested if it would be involved in the transport of anthracyclines due to its cationic properties. In this study, we defined the conditions necessary to monitor doxorubicin uptake into yeast cells, using both FACS analysis and microscope, and demonstrated for the first time that Agp2 is required for the drug uptake. The results show that doxorubicin uptake is partly dependent upon both the Dur3 and Sam3 transporters and that at least one additional permease exists in yeast cells to mediate the drug uptake. We took advantage of this model system and established that the *C*. *elegans* organic cation transporter OCT-1 has the ability to rescue doxorubicin uptake in the *agp2Δ* mutant to nearly the parental level. We created four separate amino acid substitutions within the OCT-1 protein and showed that neither one of these mutants was capable of mediating doxorubicin into the *agp2Δ* mutant. Overexpression of OCT-1 sensitized parent cells to doxorubicin, suggesting that OCT-1 is indeed a transporter of anthracyclines in higher eukaryotic cells.

## Material and Methods

### Yeast strains, media and chemicals

The *S*. *cerevisiae* wild-type strain BY4741 and its isogenic single, double and triple mutants *dur3Δ*, *sam3Δ*, *agp2Δ*, *sam3Δdur3Δ*, and *sam3Δdur3Δ agp2Δ* were used in this study [5]. The strains were grown in either rich media, i.e., yeast peptone dextrose (YPD, 1% yeast extract, 2% peptone, 2% dextrose) or minimal media (0.65% yeast nitrogen base w/o amino acid (YNB), 0.17% omission mix, and 2% dextrose). Solid media contained 2% of agar. Doxorubicin-HCl was provided by our clinical department (Maisonneuve-Rosemont Hospital (HMR), Montreal, Canada). Mouse anti-MYC antibody was a gift from Dr. Hugo Wurtele (HMR). The goat anti-mouse antibody was purchased from Enzo Life Sciences (Cat. ADI-SAB-101-J).

### Doxorubicin uptake assay

Cells were grown overnight at 30°C in either YPD or minimal media as required, the next day the cells were placed in fresh media and allowed to grow for an extra 1 h. The cultures were washed once with low YNB (i.e., minimal media with 0.065% YNB, instead of 0.65%), and 1 ml of cells of OD_600_ with absorbance of ~1.0 was centrifuged and the cell pellet was resuspended in 50 μl of the low YNB media. To this, 14.3 μl of doxorubicin-HCl (stock solution 2 mg/ml) was added to a final concentration of 800 μM. The uptake reaction was incubated for 30 min at 30°C, cells were quickly spun in an eppendorf centrifuge (10,000 rpm for 15 sec), the supernatant discarded into a biosafety container, followed by the addition of 100 μl of phosphate buffer with formaldehyde (0.367% of K_2_HPO_4_, 1.25% of KH_2_PO_4_, 3.7% of formaldehyde) to the cell pellets for 10 min at room temperature to stop the uptake reaction. Cells were briefly spun, the buffer was removed, cell pellets were resuspended in 100 μl of PBS and processed for FACS or epifluorescent microscopy (see below). For kinetic studies, cells were incubated with 0 to 1.0 mM of doxorubicin for 10 mins and processed as above.

### Cytometry analysis

Cells from the doxorubicin uptake assay were diluted in 1 ml of PBS in 5-ml polystyrene round bottom tubes and then passed through a FACS Calibur (BD Science, excitation 488 nm FL2 detector at 585/42 nm as previously described [19]. A threshold was set with a blank (i.e., cells that were not incubated with doxorubicin) and the values below this threshold were considered as zero.

### Epifluorescent microscopy

Cells (1 μl) from the doxorubicin uptake assay were placed into the wells of multitest slide 15 (MP Biomedicals) that were pre-coated with 1 mg/ml concanavalin A (MP Biomedicals). The slides were air dried completely and 1 μl of mounting medium with DAPI (UltraCruz) was added to each well. A cover glass was sealed onto the slide, images were taken with an epifluorescent microscope (Olympus B53 upright epifluorescent microscope equipped with an Olympus XM10 camera at 63X with Texas red filter to detect DOX or with the Zeiss Imager Z2 microscope equipped with Zeiss AxioCam MRC camera) and processed with Image J.

### DeltaVision microscopy

Cells (WT) were grown in YPD overnight and the next day washed, transferred into low YNB followed by treatment with 100 μM DOX for 30 min. Cells were fixed with formaldehyde as above and then attached onto concanavalin A pre-coated slides (UltiDent frosted microscope slides 170-7107A). Imaging was made with an Olympus IX71 DeltaVision Elite microscope from Applied Precision Inc. at 100x. For DOX excitation and emission, the FITC filter and mCherry were used. Images were taken with a Front Illuminated sCMOS camera and processed with ImageJ.

### RT-PCR analysis

This analysis was conducted as previously described [5].

### Construction of the pCeOCT-1 expression plasmid

The cDNA of the *C*. *elegans oct-1* gene was amplified by polymerase chain reaction (PCR) with Platinum*pfx* DNA Polymerase (Invitrogen) using the primers listed in Table 1, and a template plasmid pCeOCT1 carrying a full length cDNA that is 1826 bp long (bearing a 1707 bp long open reading frame with the termination codon) and provided by Dr. Vadivel Ganapathy (Augusta, GA, USA). The amplified PCR product was gap-repaired into the plasmid pTW438 as previously described [5]. The cDNA within the resulting plasmid pCeOCT-1 was sequenced and the plasmid was transformed into the indicated yeast strains using the lithium acetate method [20].

**Table 1:** Oligonucleotide primers used in this study.

Primers	Purpose	Sequence
pTW438-CeOCT-1-MYC–F	Gap repair into the expression plasmid pTW438	5'-CTGCACAATATTTCAAGCTATACCAAGCATACAATAAGCTTATGT CTGCAACTAAACCTCCA
pTW438-CeOCT-1-MYC–R	Gap repair into the expression plasmid pTW438	5'-GGTTACCGCAAGTCCTCTTCAGAAATGAGCTTTTGCTCGGACGCC ATGGTGAGCTAAATATTCATAAGTCGACTACT
Q15A–F	Site-directed mutagenesis	5'-AGATTTTGATTTCGTTCTAGAAGCGGTTGGCAACTATGGTACTTA TCAGA
Q15A–R	Site-directed mutagenesis	5'-TCTGATAAGTACCATAGTTGCCAACCGCTTCTAGAACGAAATCA AAATCT
C31A–F	Site-directed mutagenesis	5'-ATTGTTTTCTTCTTTATAATTGCCCTTCCAACTAGTTTACCATCAG CATT
C31A–R	Site-directed mutagenesis	5'-AATGCTGATGGTAAACTAGTTGGAAGGGCAATTATAAAGAAGAA AACAAT
Q109A–F	Site-directed mutagenesis	5'-CGAATAAGTTTAGTGCCGTGCGCAAATGGATGGGATTATGATAA CTCTAC
Q109A–R	Site-directed mutagenesis	5'-GTAGAGTTATCATAATCCCATCCATTTGCGCACGGCACTAAACTT ATTCG
K300A–F	Site-directed mutagenesis	5'-CATCAACATCAACATTTGACGCTCCATTCATCTTTGCAATTTTCTT AAGC
K300A–R	Site-directed mutagenesis	5'-AAGAAAATTGCAAAGATGAATGGAGCGTCAAATGTTGATGTTG ATGAATT

### Site-Directed mutagenesis

The QuickChange II XL Site-Directed Mutagenesis Kit from Agilent Technologies (#200521) was used according to the manufacturer`s Instruction manual Revision E.01 in order to create the amino acid substitution mutations with the primers listed in Table 1.

### Western blot

Cell pellets from 10 ml of overnight grown cells were resuspended in 200 μl of extraction buffer (50 mM Tris-HCl, 50 mM NaCl, 5% glycerol, and containing the protease inhibitor cocktail, EDTA-free (Roche) using 1 protease inhibitor tablet per 50 ml of extraction buffer), followed by the addition of 200 μl of glass beads 0.5 mm. Total extract was prepared by vortexing the samples for 20 min at 4°C. The extract was centrifuged 3 min at 3,000 rpm in an eppendorf centrifuge and the supernatant was recovered. The protein concentration in the extract was quantified using the Bio-Rad Protein Assay Dye Reagent Concentrate. Equal amounts of protein extract were mixed with loading buffer (50 mM Tris-HCl pH 6.8, 2% SDS, 0.1% bromophenol blue, 2% (v/v) 2-mercaptoethanol) and loaded onto a 8% polyacrylamide SDS gel followed by electrophoresis for 1.5 h at 100 V. Proteins were transferred onto a PVDF membrane 0.2 μm for 1 h at 100 V. After 1 h of blocking in Tris-buffer saline with Tween (TBST) (25 mM Trisbase, 2.7 mM KCl, 137 mM NaCl, 0.1% Tween-20 pH adjusted to 7.4) in 5% milk powder, the membrane was washed in TBST and then incubated overnight at 4°C in the TBST 1% BSA, 1 mM sodium azide and 1:1.000 mouse anti-MYC antibody. Next morning the membrane was washed 2 times, 5 min each in TBST, then incubated 1 h at room temperature in TBST 5% milk with 1:1000 goat anti-mouse secondary antibody. The membrane was washed 3 times, 15 min each wash with TBST. The membrane was developed using ECL by mixing solutions A and B. Solution A (0.5 ml) (100 mM Tris-HCl pH 8.5, 0.4 mM Coumaric acid dissolved in DMSO and 2.5 mM Luminol dissolved in DMSO) and solution B (0.5 ml) (100 mM TrisHCl pH 8.5, 0.018% H_2_O_2_). The polypeptide bands were detected by ImageQuant Las 4000.

### Survival assay

Cells were prepared as for the doxorubicin uptake assay described above and samples withdrawn, diluted 10,000 fold in sterile distilled water and 100 μl plated onto minimal selective media plates for cells carrying a plasmid or onto YPD media to score for survivors, as previously described [2].

## Results

### YPD and not minimal media facilitates DOX uptake into yeast cells

**Fig 1:**
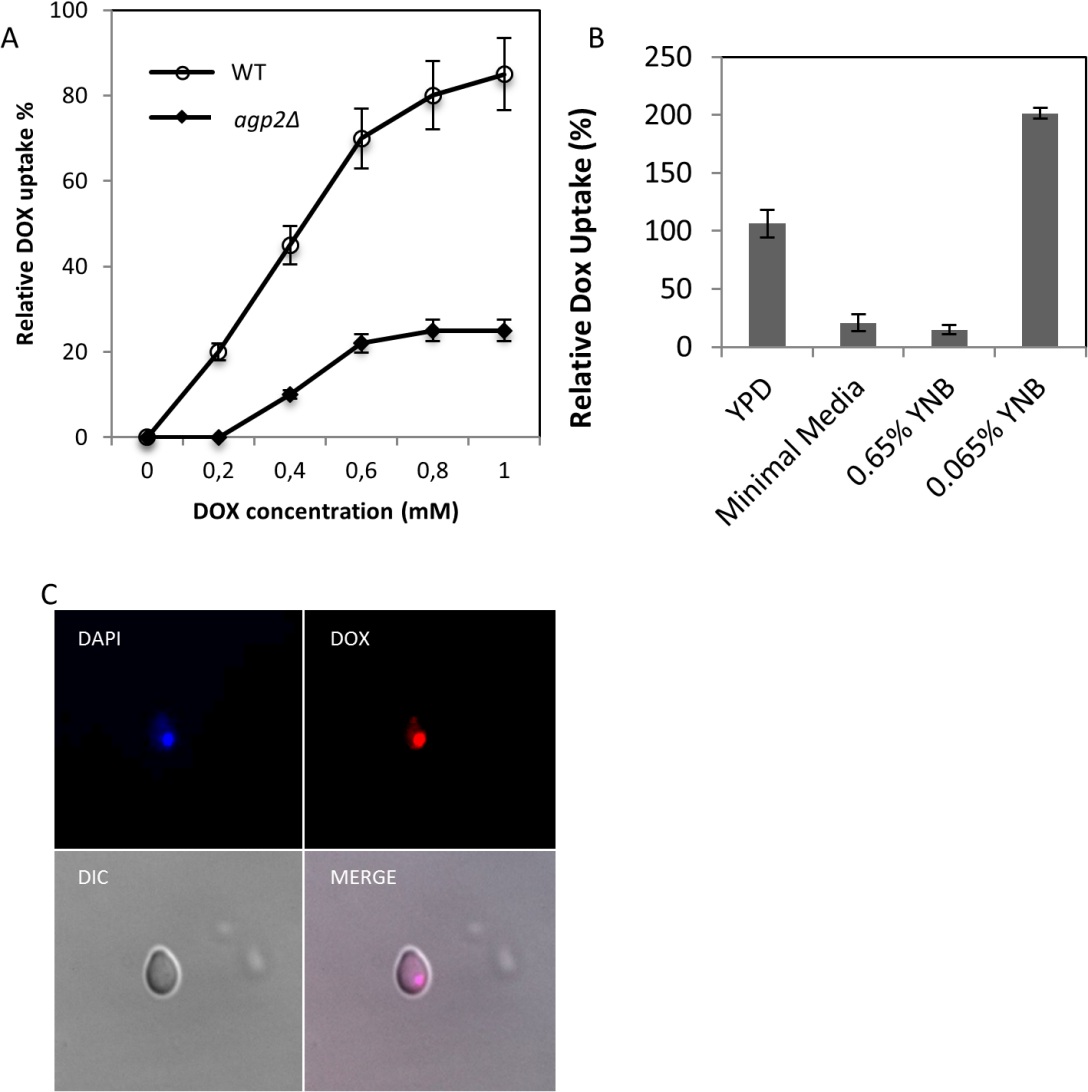
Relative DOX uptake into yeast cells in rich and minimal media, and localization of the drug to the nucleus. (A)Concentration dependent uptake of DOX into the wild type (WT) strain BY4741. Cells were grown in YPD media overnight and subcultured into the same media for 1 h followed by the addition of increasing concentration of DOX and uptake was stopped after 10 min. The intracellular accumulation of DOX was monitored using FACS analysis. The *agp2Δ* mutant defective in DOX uptake is described below. (B)Comparison of DOX uptake in YPD, minimal media, and media containing normal amount (0.65%) of yeast nitrogen base (YNB) and 10-fold less YNB (0.065%). The WT strain was incubated in the indicated media with 800 μM of DOX for 30 min, and the intracellular accumulation of the drug was measured by FACS analysis. For panels A and B, the results were the averages of three independent analyses. (C)Epifluorescent microscopy showing intracellular colocalization of DAPI and DOX in the WT strain. The WT cells grown in YPD were transferred to low YNB followed by the uptake of DOX (100 μM) for 30 min. The fixed cells were processed for microscopy using mounting medium containing DAPI to detect the nuclear DNA. Images were captured with a DeltaVision (see material and methods). DIC, differential interference contrast; Merge, colocalization of DAPI stained nucleus with DOX.

Assay conditions for monitoring the uptake of anthracyclines into yeast cells have not been previously described. To study doxorubicin (DOX) uptake, we defined the optimal uptake conditions by first testing whether intracellular accumulation of the drug could occur in the yeast growth media. We added increasing concentrations of DOX directly to yeast cultures that were in fresh yeast peptone dextrose (YPD) growth media. The DOX treatment was stopped after 10 min of incubation to assess for the drug uptake into the cells using flow cytometry (see Materials and Methods). DOX uptake into the parent strain BY4741 (WT) was readily detected in the YPD media (Fig 1A). Uptake was linear when DOX concentration was within 200 to 600 μM (Fig 1A) and only reached saturation when the extracellular concentration of the drug was approaching 1 mM. For subsequent assays, DOX was used at 800 μM and the uptake was terminated after 30 min when the drug accumulation was maximal. Since no detectable uptake was observed in the 10 μM range of DOX (Fig 1A), a concentration that is considered optimal for high affinity transporters [3], it would appear that under these conditions (800 μM for 30 min) the drug uptake is mediated by a low affinity permease.

We next conducted similar uptake analysis, but in minimal growth media. Surprisingly, when DOX was added to this media, uptake of the drug was sharply reduced (Fig 1B), even after increasing the concentration of the drug to 1.5 mM and extending the incubation period to 60 min. A simple interpretation of this observation is that the minimal media may contain constituents that block DOX uptake. The minimal media is composed of a mixture of amino acids, dextrose, and yeast nitrogen base (YNB) that includes vitamins and divalent metal ions. We examined whether elimination of the amino acids and or dextrose from the minimal media would allow DOX uptake into the cells. Removal of either the amino acids or dextrose or both simultaneously and keeping only the YNB (0.65% YNB) did not permit uptake of DOX into the cells, eliminating the possibility that the amino acids and dextrose act to block DOX uptake (Fig 1B). However, when DOX uptake was monitored in the minimal media where the composition of only YNB was reduced by 10-fold (0.065% YNB referred to as low YNB), drug uptake was strikingly rescued and leading to nearly 2-fold stimulated uptake as compared to YPD (Fig 1B). Similarly, removal of the amino acids and dextrose from the low YNB did not alter the level of DOX uptake, suggesting that uptake could be inhibited by one or more components present in the standard concentration of YNB. As such, we used only the low YNB media as the standard assay conditions to monitor DOX uptake.

We examined whether the uptake of DOX would lead to the accumulation of the drug in the nucleus. Indeed, epifluorescent microscopy revealed that following DOX uptake (low YNB with 100 μM DOX) the drug, which has a known property of autofluorescing at 640 nm, accumulated in the nucleus since staining of the nuclear DNA with DAPI coincided with the staining for DOX as shown by the resulting merged image (Fig 1C, showing a single cell). This observation is consistent with the mechanism of action of DOX on DNA. It is noteworthy that when DOX uptake was performed at concentrations > 100 μM, the drug severely compromised staining of the nuclear DNA with DAPI (data in S1 Fig). We suggest that intercalation of DOX with the DNA interferes with DAPI binding.

### Divalent metal ions in the minimal media inhibit DOX uptake

**Fig 2:**
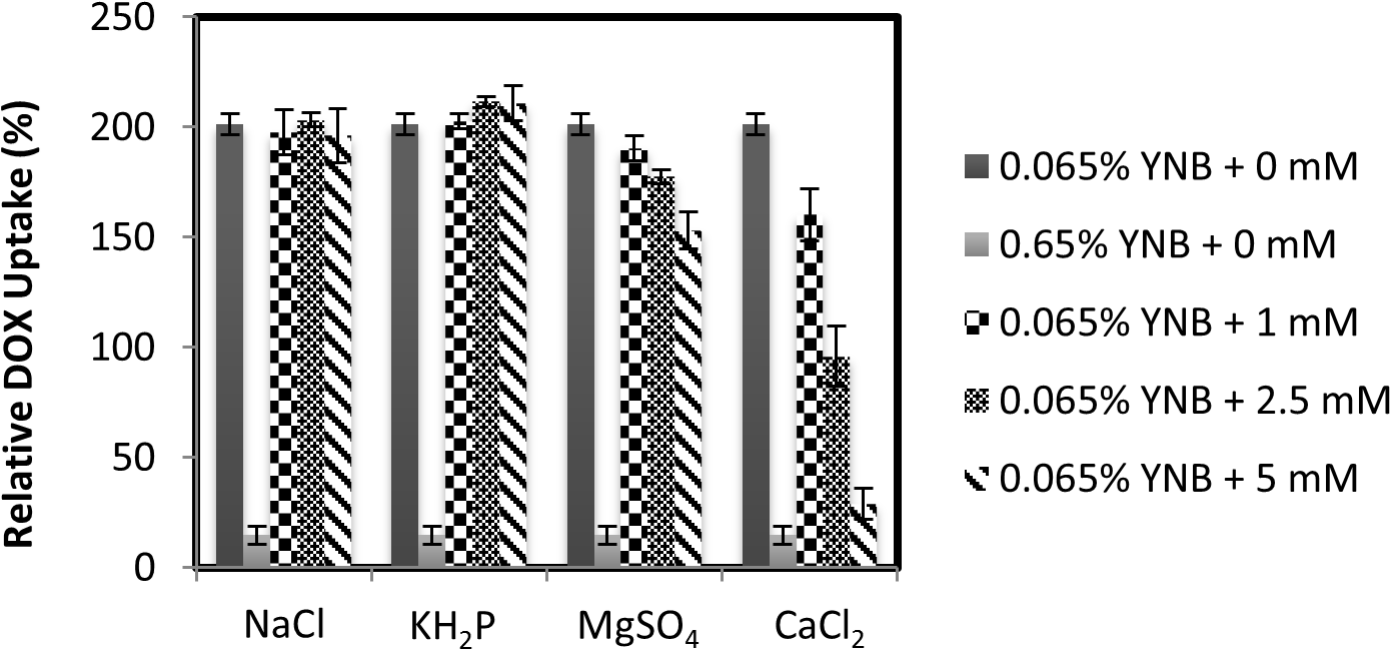
Divalent metal ions present in yeast nitrogen base inhibit DOX uptake into the cells. The WT strain grown in YPD was washed, resuspended in either low YNB or standard YNB. Uptake assay was started by the addition of 800 μM of DOX and, at the same time, without and with increasing concentrations of the indicated salts (0 to 5 mM) to the cells in low YNB. Uptake reaction was stopped after 30 min and the intracellular accumulation of DOX was measured by FACS analysis. The results were the averages of three independent experiments.

The YNB contains various vitamins, as well as monovalent and divalent salts, namely NaCl, CaCl_2_, MgSO_4_, K_2_HPO_4_ and KH_2_PO_4_, although the exact concentration of these components are not revealed by the manufacturer. We assessed whether addition of increasing concentrations of these salts would interfere with the uptake of DOX in the low YNB. Addition of NaCl, K_2_HPO_4_ or KH_2_PO_4_ to the low YNB did not block the uptake of DOX into the yeast cells (Fig 2). Interestingly, CaCl_2_ at 5 mM potently inhibited the uptake of DOX into the cells, while MgSO_4_ was less effective at the same concentration (Fig 2). Other divalent metal ions such as Zn^2+^ and Mn^2+^ also inhibited DOX uptake, but this occurred at substantially higher concentrations unlikely to be present in the minimal media. As such, we strongly suggest that the high concentration of divalent cations such as Ca^2+^ in the minimal media is suppressing the uptake of DOX into the cells (see discussion). However, addition of CaCl_2_ alone to YPD media did not block DOX uptake (data not shown), suggesting that CaCl_2_ acts together with additional factors and contribute to the inhibition of DOX uptake in minimal media.

### Yeast mutants defective in polyamine uptake are also defective in DOX uptake

**Fig 3:**
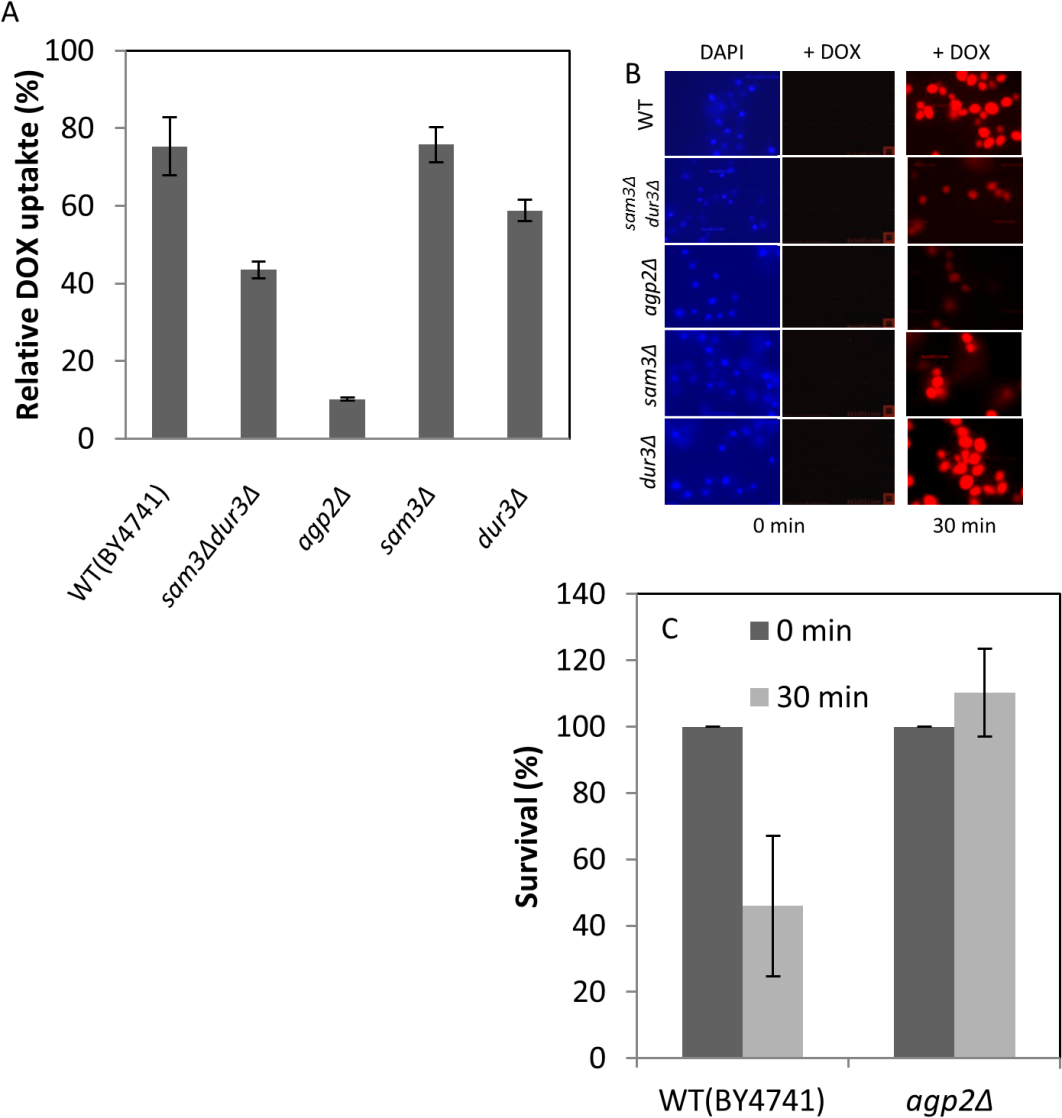
Mutants lacking Agp2 are deficient in DOX uptake and display resistance to the drug. (A) FACS analysis of DOX uptake levels in the WT and the isogenic mutant strains. Cells were incubated in low YNB in the presence of DOX (800 μM) for 30 min and processed for FACS analysis to quantify the level of drug uptake. The background uptake detected in the presence of normal amount of YNB, which vary between 10 to 20% was subtracted from each analysis to provide the real intracellular uptake of DOX. (B) Epifluorescent microscopy showing the relative accumulation of DOX in the WT and the isogenic mutants. Uptake of DOX (800 μM) for 30 min was performed in low YNB and the cells processed for epifluorescent microscopy. Images were captured with an Olympus epifluorescent microscope equipped with a camera (see materials and methods). (C) Comparison of the surviving fractions of the WT and *agp2Δ* mutant following exposure to DOX. Uptake was conducted as in panel A, cells diluted and plated for survivors on solid YPD. The results were the averages of two independent experiments.

We previously documented that Agp2 is a regulator that controls the expression of several plasma membrane transporters [5]. Cells devoid of Agp2 showed resistance to several cationic compounds such as polyamine, bleomycin, and NaD1 [2, 3, 6]. Since DOX is a cationic drug, we examined if *agp2Δ* mutant would be defective in its uptake. While the WT cells efficiently took up DOX in the low YNB media, the *agp2Δ* mutant was defective in the drug uptake (Fig 3A, see also Fig 1A in YPD), consistent with the notion that DOX uptake is dependent upon an active influx transporter regulated by Agp2. The *agp2Δ* mutant was defective in DOX uptake at all concentrations tested (see Fig 1A), but not completely as compared to the WT (Figs 1A and 3A), raising the possibility that a redundant transporter for DOX remains functional in the *agp2Δ* mutant. Using epifluorescent microscopy, we examined whether the uptake of the drug into the WT and the *agp2Δ* mutant would correlate with the FACS analysis. As shown in Fig 3B, DOX accumulation in the WT cells was significantly more intense than the *agp2Δ* mutant consistent with the FACS data. As mentioned above, and under these conditions DOX uptake severely compromised staining of the nuclear DNA with DAPI, perhaps a result of DOX intercalating with the DNA that prevented DAPI binding (data in S1 Fig.). Of importance, not all the cells from the *agp2Δ* mutant were defective in DOX uptake, as in any given field there is a fraction that take up the drug (Fig 3C). We suggest this could be the result of another transporter (see discussion).

The increased influx of DOX into the cells is expected to damage the genome and lead to cell death. We examined the surviving fraction of the WT and the *agp2Δ* mutant cells following exposure to DOX. Briefly, exponentially growing cultures in YPD were washed twice in low YNB and incubated with DOX (800 μM) for 30 min followed by plating of the diluted cells to score for the fraction of the cells that survivored the treatment. At least 45% of the WT cells did not survive exposure to the DOX treatment, while all the cells of the *agp2Δ* mutant survived (Fig 3C). We conclude from these data that the genotoxic effects of DOX depend on Agp2 for the efficient uptake of the drug into the cells.

We next tested if the known polyamine transporters Dur3 and Sam3 that are regulated by Agp2 are involved in the uptake of DOX. The *sam3Δ* mutant was as proficient as the WT in DOX uptake, while *dur3Δ* mutant showed slight decrease in the drug uptake as monitored by FACS analysis (Fig 3A). However, uptake was reduced by nearly 35 to 40% in the *sam3Δdur3Δ* double mutant where both the *SAM3* and *DUR3* genes were deleted (Fig 3A), suggesting that these transporters play a role in the uptake of DOX. The fact that DOX uptake was not completely diminished by the *sam3Δdur3Δ* double mutant is in agreement with the existence of additional permease(s) involved in the transport of this drug. It is noteworthy that the uptake of DOX by the *sam3Δdur3Δ* double and the *sam3Δ* and *dur3Δ* single mutants correlated with the level of accumulation of the drug in the cells as observed by epifluorescent microscopy (Fig 3B).

### Expression of either Dur3 or Sam3 rescues DOX uptake in the *sam3Δdur3Δ* double mutant

**Fig 4:**
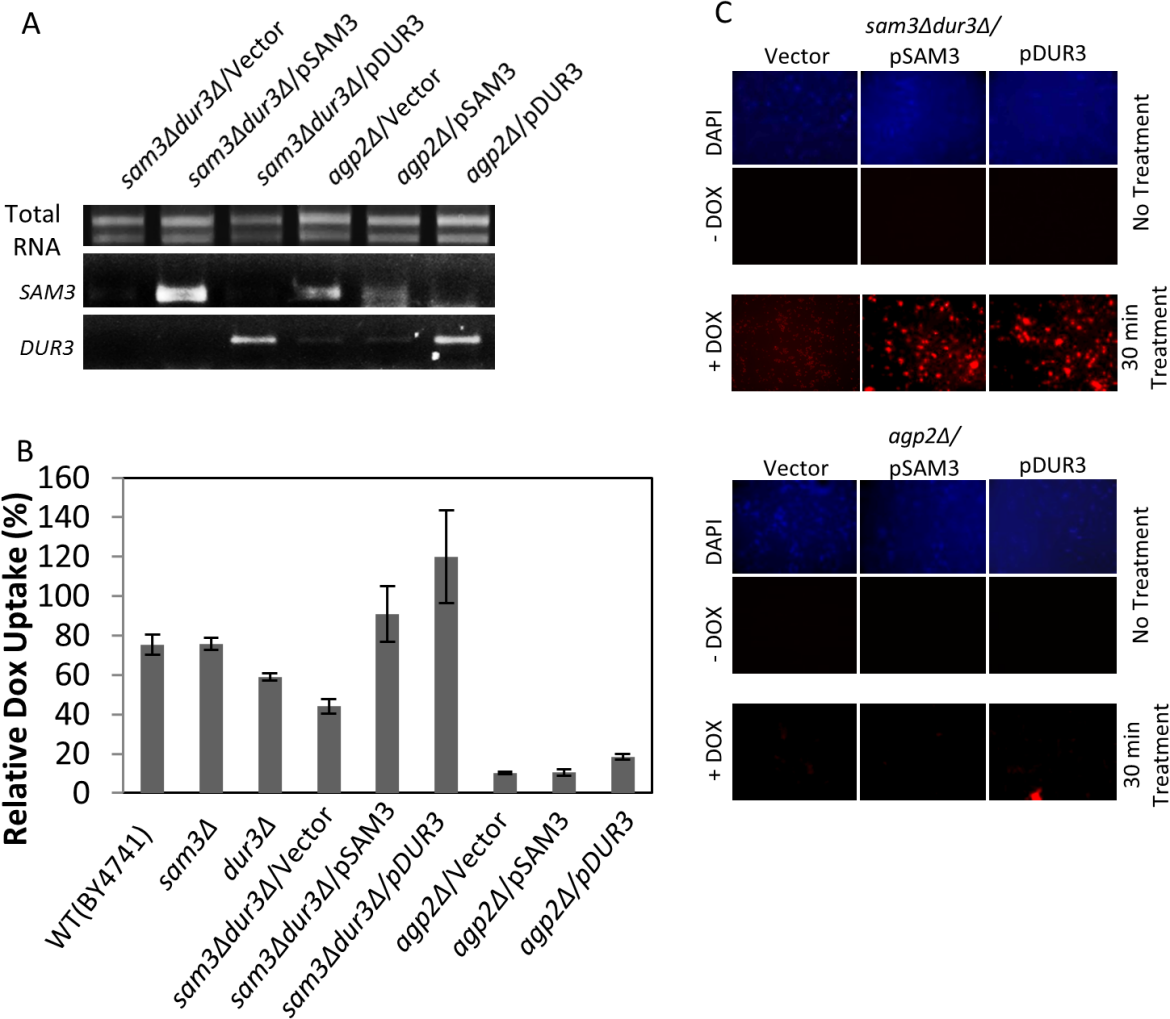
A multicopy plasmid carrying either the entire *SAM3* or *DUR3* gene rescues DOX uptake in the *sam3Δdur3Δ* double mutant but not in the *agp2Δ* single mutant. (A) RT-PCR analysis showing the expression level of *SAM3* and *DUR3* in the *sam3Δdur3Δ* and the *agp2Δ* mutants with the indicated plasmids. Total RNA (1 μg) was reverse-transcribed and the expression level of either SAM3 or DUR3 was assessed from the resulting cDNA using gene specific primers [5]. (B) DOX uptake is mediated by the Sam3 and Dur3 transporters and depends on Agp2 function. The multicopy plasmid pSAM3 or pDUR3 carrying the entire *SAM3* or *DUR3* gene, respectively, with the endogenous promoter was introduced into either the *sam3Δdur3Δ* double mutant or the *agp2Δ* single mutant and the resulting transformants monitored for DOX uptake in low YNB using FACS analysis. (C) Epifluorescent microscopy showing that either pSAM3 or pDUR3 restores DOX uptake to the *sam3Δdur3Δ* double mutant, but not to the *agp2Δ* mutant. Microscopy was conducted as in Fig 3B.

To further test if Sam3 and Dur3 are involved in DOX uptake, we examined whether expression of these transporters from their endogenous promoter and carried by a multicopy plasmid, pSAM3 and pDUR3, would rescue drug uptake in the *sam3Δdur3Δ* double mutant. RT-PCR analysis revealed that the *SAM3* and *DUR3* genes were expressed in the *sam3Δdur3Δ* double mutant when compared to the vector control (Fig 4A). Expression of pSAM3 restored DOX uptake in the double mutant to the WT level, while pDUR3 caused a modest stimulation in the uptake as monitored by both FACS and epifluorescent analyses (Fig 4B and 4C, respectively). These data provide further evidence that both Sam3 and Dur3 possess the ability to transport DOX into yeast cells. It is noteworthy that introduction of either pSAM3 or pDUR3 into the *agp2Δ* mutant did not rescue DOX uptake (Fig 4B and 4C), even though *DUR3* was expressed at comparable level when introduced into the *sam3Δdur3Δ* double mutant (Fig 4A and 4B). While we have shown previously that Agp2 is required to maintain expression of the endogenous genes, *SAM3* and *DUR3*, and which apparently could be bypassed by a multicopy plasmid as in the case for *DUR3* (Fig 4A), there is no evidence whether Agp2 is also involved in the post-transcriptional or-translational regulation of these transporters [5].

### Expression of *C*. *elegans* OCT-1 rescues DOX uptake in the *agp2Δ* mutant

**Fig 5:**
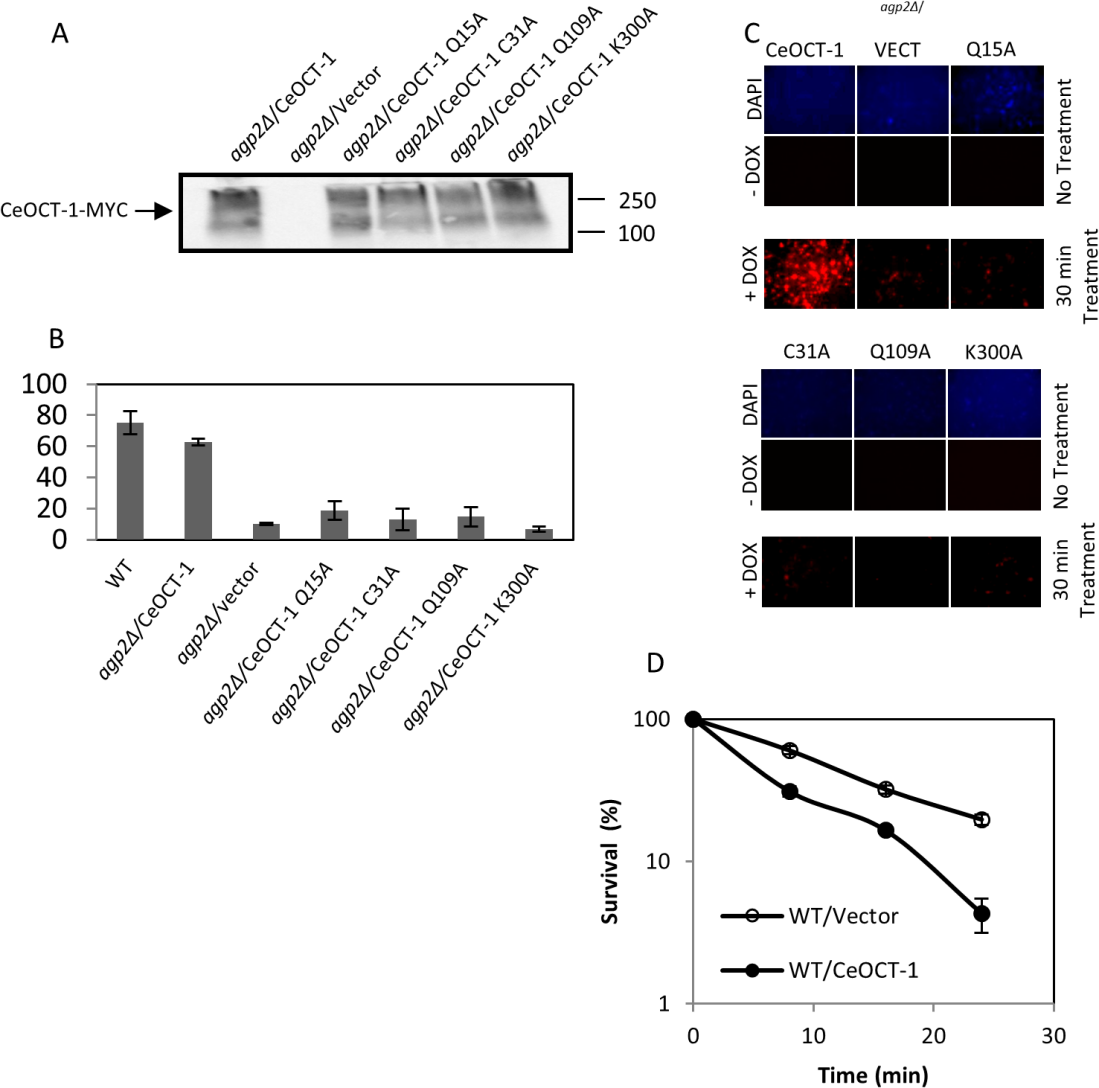
Expression of the native CeOCT-1, but not the variants, rescues DOX uptake in the *agp2Δ* mutant. Briefly, the *C*. *elegans oct-1* gene was cloned next to the *ADH* promoter and placed in frame with a C-terminal MYC epitope tag in the vector pTW438 to produce the plasmid CeOCT-1. The four variants were derived from pCeOCT-1 by site-directed mutagenesis. (A) Western blot analysis showing expression of CeOCT-1-MYC and its variants in the *agp2Δ* mutant. Equal amounts of total protein extracts (50 μg) were probed with anti-MYC antibodies and the molecular size markers are indicated in kD. (B) FACS analysis showing that pCeOCT-1, but not the variants, stimulates DOX uptake to nearly WT level in the *agp2Δ* mutant. DOX uptake was monitored using FACS analysis. (C) Epifluorescent microscopy showing that pCeOCT-1, but not the variants, causes the accumulation of DOX in the *agp2Δ* mutant. The cells used for this analysis were the same as for the FACS analysis in panel B. (D) Expression of CeOCT-1 sensitizes the WT cells to the killing effects of DOX. Exponentially growing cells in selective minimal media were washed twice with the low YNB uptake buffer, adjusted to OD600 of ~ 1.0 then incubated in the same buffer with 800 μM DOX in a final volume of 100 μl. Samples 20 μl were taken at 0, 8, 16, and 24 mins, diluted 10,000 fold and 100 μl plated onto solid selective minimal media to score for the surviving factions, expressed as a percentage of the zero time point set at 100%.

Our next objective was to determine whether higher eukaryotic cells carry genes that will permit uptake of DOX into yeast cells. Since Sam3 and Dur3 can transport a variety of cationic compounds, in particular, the prototypical substrate tetraethylammonium (TEA) used for classifying transporters into organic cation transporter family, we decided to search the literature for higher eukaryotic transporters with the ability to transport [^14^C]-labeled TEA [21]. The search revealed the *C*. *elegans* OCT-1 (CeOCT-1) protein identified as a transporter for TEA and more recently as a transporter for ergothioneine [21, 22]. Analysis from the wormbase sequence data revealed that there are two isoforms, OCT-1a and OCT-1b, with OCT-1a possessing an extra 17 amino acid residues at the N-terminus (MSFQAMETFAEISQEIL) as compared to OCT-1b (data in S2 Fig). Deletion of the *oct-1* gene in *C*. *elegans* has been shown to shorten the lifespan of the animal, which may be related to oxidative stress caused by a defect in the import of the antioxidant ergothioneine [22]. Comparison between CeOCT-1 and Sam3 or Dur3 revealed no significant identity as determined by the CLUSTAWL program, but CeOCT-1 shared 31.1% identity with the human OCT1 (data in S2 Fig). We obtained a cDNA clone, which upon DNA sequencing corresponded to the *F52F12*.*1* gene locus of chromosome 1 encoding the CeOCT-1b isoform [21]. We engineered a construct pCeOCT-1 to express CeOCT-1b in the yeast *agp2Δ* mutant using gap repair such that the expression was driven by the yeast constitutive *ADH* promoter and carrying a C-terminal MYC tag. As shown in Fig 5A, the CeOCT-1-MYC fusion protein was expressed in the *agp2Δ* mutant as monitored by Western blot analysis probed with anti-MYC monoclonal antibody. The expected size of CeOCT-1 is approximately 62 kDa and with the MYC tag the predicted size is estimated to be 64 kDa. However, expression of CeOCT-1 in yeast generated a protein that was substantially higher in molecular weight, suggesting that the protein is likely modified in yeast cells causing a significant shift in its mobility. In fact, CeOCT-1 is predicted to have three potential *N*-glycosylation sites Asn-70, Asn-81, and Asn-116 [21].

We next examined whether CeOCT-1 expression would stimulate DOX uptake in yeast cells. The expression of CeOCT-1 in *agp2Δ* mutant stimulated DOX uptake by 6-fold when compared to the mutant carrying the empty vector, which was assessed by both FACS and epifluorescent analyses (Fig 5B and 5C). The level of DOX uptake stimulated by CeOCT-1 expression in the *agp2Δ* mutant was nearly comparable to the level of drug uptake observed in the WT strain (Fig 5B). This suggests that expression of CeOCT-1 can function to take up DOX into yeast cells, but it is unable to further stimulate uptake beyond the level observed in the WT cells. We note that the *ADH* promoter driving the expression of CeOCT-1 is independent of Agp2 function [5].

### The *C*. *elegans* OCT-1 variants Q15A, C31A, Q109A and K300A are expressed, but defective in rescuing DOX uptake in the *agp2Δ* mutant

To confirm that CeOCT-1 is indeed responsible for DOX uptake in the *agp2Δ* mutant, we examined the effect of four separate amino acid substitutions Q15A, C31A, Q109A and K300A within the transporter. We created these CeOCT-1 variants as the substituted amino acid residues are conserved in the human OCT1 transporter (data in S2 Fig). In addition, we wanted to test whether changing the amino charge in different regions that include the N-terminal, transmembrane, extracellular and intracellular domains would interfere with the protein function [21]. The CeOCT-1 variants were expressed under the same expression system as the native CeOCT-1. All four CeOCT-1 variants, CeOCT-1-Q15A, CeOCT-1-C31A, CeOCT-1-Q109A, and CeOCT-1-K300A were expressed at similar level and with the same high molecular weight forms as the native CeOCT-1 protein when monitored by Western blot probed with anti-MYC (Fig 5A). These four variants were all defective in DOX uptake into the *agp2Δ* mutant, as compared to the native CeOCT-1 protein (Fig 5B and 5C). Since single mutations blocked the transport function of CeOCT-1, it appears that CeOCT-1 acts directly, and not via an interaction with another yeast transporter, to trigger the influx of DOX into the cells.

### Expression of CeOCT-1 sensitizes the WT strain to the genotoxic effects of DOX

Since CeOCT-1 mediated the transport and accumulation of DOX into the yeast cells, we reasoned that the expression of this transporter would enhance the sensitivity of cells to the drug. In order to verify this hypothesis, the vector and the pCeOCT-1 plasmid were separately introduced into the WT cells and the resulting transformants were tested for DOX sensitivity by scoring for survivors. Briefly, exponentially growing cultures in minimal media were washed twice in low YNB and incubated with DOX (800 μM) followed by plating the cells to score for the survivors. When the WT cells expressing CeOCT-1 were challenged with DOX there was a time-dependent decreased in cell survival with only 5% of the cells surviving after 24 min (Fig 5D). In contrast, substantially more cells, ~ 35 to 40%, that carried only the empty vector survived the treatment for 24 min (Fig 5D). Collectively, our data suggest that transporters exist in eukaryotic cells that can regulate cellular response to anthracyclines.

## Discussion

In this study, we show for the first time the yeast plasma membrane transporters Sam3 and Dur3, as well as their regulator Agp2, are involved in mediating the uptake of anthracyclines into the cells. These findings negate earlier claims that anthracyclines enter cells by simply diffusion across the plasma membrane (see in review [23]). Both Sam3 and Dur3 are not specific for anthracyclines as they serve to transport other substrates including polyamines into the cells [4, 5]. Our data suggest that the Agp2-regulated transporters operate with low affinity, mediating uptake when the drug concentration is high, that is, in excess of 100 μM. However, we cannot rule out the possibility that the assay conditions developed herein might be suboptimal and therefore it might not accurately assess the uptake affinities for these transporters. Many organisms including yeast have both low and high affinity transporters for various substrates such as polyamines, potassium, calcium, and amino acids, indicating that there might also be a high affinity DOX transporter in yeast [3, 5, 24]. We did not observe uptake at low DOX concentrations (10 μM) and perhaps the assay conditions might not be suitable to detect the existence of a high affinity transporter for DOX. Our observations that deletion of both *DUR3* and *SAM3* did not completely block DOX uptake and that the deletion of *AGP2* only further reduced the uptake, raise the possibility that at least one additional transporter exists to take up DOX into the cells. In fact, a *dur3Δsam3Δagp2Δ* triple mutant showed the same low level of DOX uptake as the *agp2Δ* single mutant (data not shown), excluding the possibility that Dur3 and/or Sam3 could be the redundant transporter in the *agp2Δ* mutant. It is therefore possible that the existence of redundant DOX transporters might have prevented their detection in previous studies that applied genome-wide screens [9, 25].

In is noteworthy that in the case of the *agp2Δ* mutant there are isolated cells in the population that showed significant DOX staining by epifluorescent microscopy as if these cells efficiently took up the drug (data in S1 Fig.). These brightly stained cells showed normal morphology and do not appear to be dead cells by FACS analysis, which would give a specific FSC/SSC location. As such, we speculate that these cells could be a reflection of a subpopulation expressing a redundant transporter. We have not attempted to synchronize the *agp2Δ* mutant and measure DOX uptake at the different phases of the cell cycle to test if the subpopulation exists in a particular phase. Nonetheless, we predict that deletion of this putative transporter in the *agp2Δ* mutant should completely abolish the drug uptake.

Herein, we found that CaCl_2_ is a potent inhibitor blocking DOX uptake into yeast cells when the assay was conducted in minimal, but not in YPD, media. It is unlikely that Ca^2+^ binds to DOX to form a complex that impedes the drug uptake, as addition of CaCl_2_ to the uptake assay system for anthracyclines into mammalian cells did not prevent the drug entry (E.M., N.B., and D.R., in preparation). It is also unlikely that CaCl_2_ shut down the synthesis of Dur3, Sam3 and other transporters responsible for DOX uptake, as these transporters have not been implicated in preventing Ca^2+^ toxicity and the *agp2Δ* mutant is no more resistant to CaCl_2_ than the parent [2, 5]. Moreover, we further exclude the possibility that CaCl_2_ stimulates rapid efflux of DOX from the cells, as cells pre-incubated with DOX for 30 min followed by the addition of CaCl_2_ did not interfere with the intracellular level of the accumulated DOX. A reasonable explanation for the DOX uptake inhibition by the elevated level CaCl_2_ is that Ca^2+^ may signal a stress response that either inactivates the function of the transporters or activates the cell wall integrity pathway such that it alters the composition of the cell wall to prevent passage of the drug when cells are incubated in the minimal media [26]. In yeast, elevated concentrations of Ca^2+^ is sensed by the Ca^2+^ sensor protein calmodulin, which binds and activates the protein phosphatase calcineurin [27, 28]. Calcineurin can act to dephosphorylate the transcription factor Crz1, which then translocates from the cytosol to the nucleus where it binds to promoters with calcineurin-dependent response element to activate expression of nearly 160 genes some of which encode proteins that are involved in cell wall integrity [27–29]. Thus, in the presence of high Ca^2+^, diffusion of DOX across the cell wall might be blocked and preventing it from reaching the plasma membrane. Alternatively, since Dur3 is phosphorylated, and possibly Sam3, by the Ptk2 kinase [4], it seems possible that the Ca^2+^ activated calcineurin phosphatase could dephosphorylate these transporters and inactivate the uptake functions. Testing these possibilities would require extensive genetic studies that disrupt components of the Ca^2+^ signaling and the cell wall integrity pathways.

The restoration of DOX uptake into the *agp2Δ* mutant by expression of the *C*. *elegans* OCT-1 transporter strongly suggests that the transporters, as well as the substrate specificities, are conserved across species. However, we could not test directly whether CeOCT-1 functions as a DOX transporter in *C*. *elegans*, since uptake measurements are complicated by endogenous autofluorescent molecules with the same emission spectrum as DOX. Nonetheless, our data suggest that the yeast model system is useful to search for similar uptake permease(s) in mammalian cells. Preliminary uptake studies revealed that DOX can be actively transported into several human cell lines (E.M., N.B., and D.R., in preparation), although the nature and identity of the transporter await further studies. It is noteworthy that an earlier study by Okabe et al., claimed that the organic cation transporter hCT2 is an uptake transporter for DOX [18]. We have expressed hCT2 in our yeast system, but unable to detect hCT2-mediated DOX uptake. There are at least five other organic cation transporters OCTN1, OCTN2, OCT1, OCT2 and OCT3, that have been characterized for functional roles in the uptake of various substrates that include L-carnitine, choline, ergothioneine, and the diabetes and anticancer drugs, metformin and imatinib, respectively [30, 31][32–34]. The *C*.*elegans* OCT-1 shares varying homology and distinct differences with each of these human organic cation transporters, for example, CeOCT-1 shares nearly 31% identity in a stretch of 440 amino acid residues with the human OCT1 (data in S2 Fig.), and thus it is possible that more than one of these human transporters could be involved in DOX uptake. Finding a DOX transporter(s) would be of great advantage as it might help to determine whether patients who are resistant to DOX treatment possess a defect in the drug uptake and thus would benefit from alternative therapy, as opposed to performing induction therapy without the knowledge whether the drug can enter or not into the cancer cells.

In short, we have documented that yeast cells contain plasma membrane transporters that mediated the uptake of an important family of anticancer drugs, anthracyclines. Identifying and characterizing these transporters in human cells are likely to provide helpful hints to explain why a significant fraction of older patients with acute myeloid leukemia is resistant to anthracyclines possibly due to defects in the drug uptake.

## Supporting Information

S1 Fig Doxorubicin compromises DAPI staining. Cells were grown in YPD media and uptake was carried out for 30 min with 800 μM DOX in either the same media or transfer to low YNB. Cells without DOX or after uptake were staining with DAPI before microscopy. In all experiments, DOX uptake severely compromised staining of the nuclear DNA with DAPI, which is likely due to the binding of DOX onto the DNA prevents DAPI binding.
(TIF)

S2 Fig Comparison of the similarities and differences amongst three organic cation transporters, *S. cerevisiae* Sam3, *C. elegans* OCT-1 and human OCT1. The predicted amino acid sequences for the transporters were obtained from NCBI and aligned using CLUSTAWL. The residues shown in red are present in the CeOCT-1a isoform, identities of 3 or more amino acid residues are highlighted in cyan, and yellow indicates distinct differences between CeOCT-1 and human OCT1. Asterisks indicate identical amino acid residues.
(TIF)
