# Supplementary figures and images for: Complementation of the Yeast Model System Reveals that Caenorhabditis elegans OCT-1 Is a Functional Transporter of Anthracyclines
Source: PLoS One. 2015 Jul 15;10(7):e0133182. doi: 10.1371/journal.pone.0133182 (PMC4503637; doi:10.1371/journal.pone.0133182)

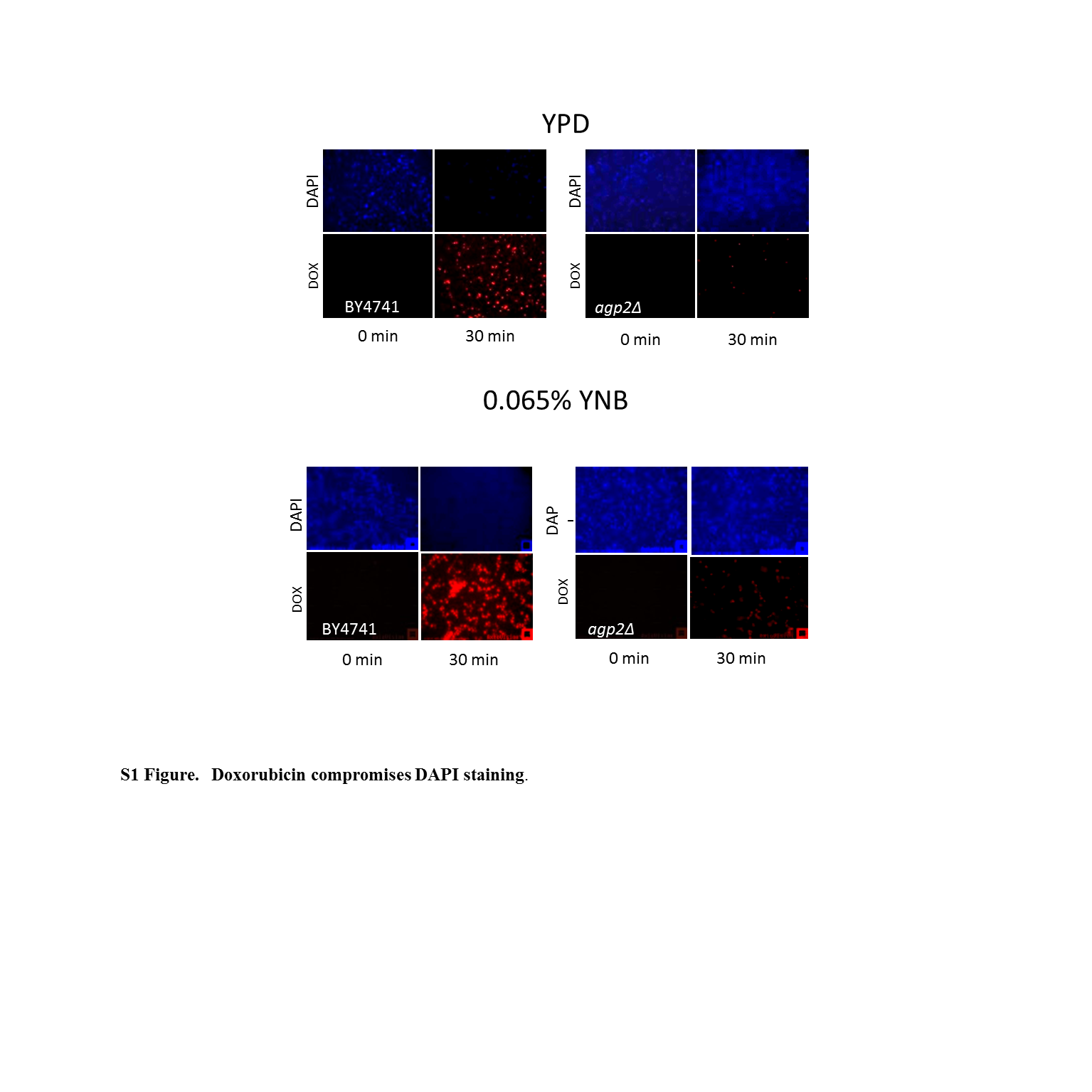

Supplement: S1 Fig — Cells were grown in YPD media and uptake was carried out for 30 min with 800 μM DOX in either the same media or transfer to low YNB. Cells without DOX or after uptake were staining with DAPI before microscopy. In all experiments, DOX uptake severely compromised staining of the nuclear DNA with DAPI, which is likely due to the binding of DOX onto the DNA prevents DAPI binding. (TIF) [file pone.0133182.s001.TIF]

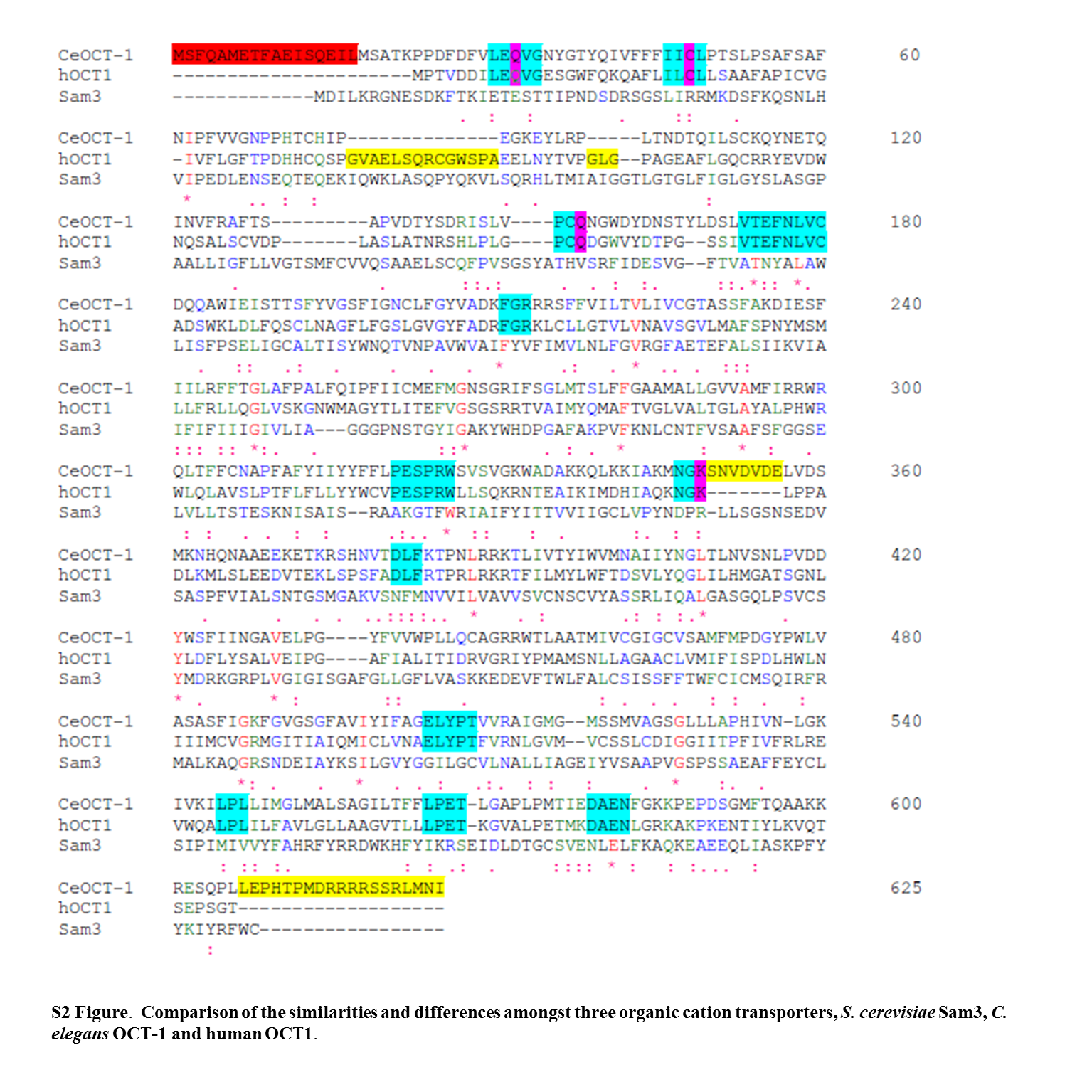

Supplement: S2 Fig — The predicted amino acid sequences for the transporters were obtained from NCBI and aligned using CLUSTAWL. The residues shown in red are present in the CeOCT-1a isoform, identities of 3 or more amino acid residues are highlighted in cyan, and yellow indicates distinct differences between CeOCT-1 and human OCT1. Asterisks indicate identical amino acid residues. (TIF) [file pone.0133182.s002.TIF]
